# PTBP3 contributes to the metastasis of gastric cancer by mediating CAV1 alternative splicing

**DOI:** 10.1038/s41419-018-0608-8

**Published:** 2018-05-11

**Authors:** Xin Liang, Weixia Chen, Haiyang Shi, Xiangyu Gu, Yueqi Li, Yingxue Qi, Ke Xu, Aiguang Zhao, Jianwen Liu

**Affiliations:** 1 State Key Laboratory of Bioreactor Engineering and Shanghai Key Laboratory of New Drug Design, School of Pharmacy, East China University of Science and Technology, Shanghai, PR China; 2 Department of Oncology, Longhua Hospital, Shanghai University of Traditional Chinese Medicine, 725 South Wanping Road, 200032 Shanghai, PR China; 3 Central laboratory, General Surgery, Putuo Hospital, and Interventional Cancer Institute of Chinese Integrative Medicine, Shanghai University of Traditional Chinese Medicine, 200062 Shanghai, PR China

## Abstract

Polypyrimidine tract-binding protein 3 (PTBP3) is an essential RNA-binding protein with roles in RNA splicing, 3′ end processing and translation. Although increasing evidence implicates PTBP3 in several cancers, its role in gastric cancer metastasis remains poorly explored. In this study, we found that PTBP3 was upregulated in the gastric cancer tissues of patients with lymph node metastasis. Patients with high PTBP3 expression levels had significantly shorter survival than those with low PTBP3 expression. Overexpression/knockdown of PTBP3 expression had no effect on proliferation, whereas it regulated migration and invasion in vitro. In addition, when a mouse xenotransplant model of MKN45 was established, knockdown of PTBP3 in MKN45 cells caused the formation of tumours that were smaller in size than their counterparts, with suppression of tumour lymphangiogenesis and metastasis to regional lymph nodes. Furthermore, we identified caveolin 1 (CAV1) as a downstream target of PTBP3. RNA immunoprecipitation (RIP) assays and dual-luciferase reporter gene assays indicated that PTBP3 interacted with the CU-rich region of the *CAV1* gene to downregulate CAV1α expression. Knockdown of CAV1α abrogated the reduction of FAK and Src induced by PTBP3 knockdown. In summary, our findings provide experimental evidence that PTBP3 may function as a metastatic gene in gastric cancer by regulating CAV1 through alternative splicing.

## Introduction

Gastric cancer is one of the most common malignancies and a leading cause of cancer death worldwide^1^. Despite great progress in surgical treatment, patients with metastatic disease have fewer treatment options, and metastasis is the major cause of mortality among patients with solid tumours^2^. The metastatic process includes detachment of tumour cells from the primary tumour mass, microinvasion of tumour cells into stromal tissues, intravasation of tumour cells into the lymphatic or blood vessels, and extravasation and growth of tumour cells in secondary sites^3, 4^. The capacity of tumour cells to induce lymphangiogenesis may determine the probability of lymphatic metastasis^5^.

Polypyrimidine tract-binding protein 3 (PTBP3) is an essential RNA-binding protein with roles in RNA splicing, 3′ end processing and translation^6–9^. PTBP3 plays an important role in regulating gene expression through a multitude of RNA-binding proteins that affect the biological behaviour of carcinoma cells^10^. Alternative splicing allows multiple mRNA isoforms to be produced by a single gene. These gene isoforms commonly encode distinct protein isoforms, thereby enriching the repertoire of proteins available to organisms^11–14^. PTBP3 expression varies widely among different cell types: it is expressed in several embryonic cell types and in adult haematopoietic cells^15^. Moreover, accumulated evidence concerning PTBP3 in different cancer types has been reported. Hepatitis B virus core protein inhibits Fas-mediated apoptosis of hepatoma cells via the regulation of mFas/FasL and sFas expression by increasing Fas alternative mRNA splicing^16^, and the cell motility of lung adenocarcinoma cells was impaired by knocking down PTBP3 expression^17^. In addition, PTBP3 expression is upregulated in gastric cancer compared with that in normal gastric mucosa, a finding that may closely positively correlate with gastric carcinoma progression^18^.

However, the role of PTBP3 in gastric carcinoma tumour formation and metastasis remains poorly understood. Here, to gain better insight into the function of PTBP3 in gastric carcinoma progression, we used MKN45 gastric cells as a major experimental system to directly explore the function of PTBP3 in gastric cancer tumour growth and metastasis.

Caveolin 1 (CAV1) is an integral membrane protein that localises to multiple cellular domains^19^ and whose expression during tumourigenesis is stage specific and compartmentalised. Studies have revealed that CAV1 has two different transcription isomers: CAV1α and CAV1β^20^. Only CAV1α (wild-type CAV1) has the Tyr-14 region at the *N* terminus. CAV1α promotes cell death and inhibits tumour metastasis, whereas CAV1β promotes cell survival, suggesting opposite biological functions of CAV1α and CAV1β^21–23^. CAV1 has been reported to be regulated by PTBP3 though alternative splicing by directly binding to CAV1 mRNA^10^. Thus, a question remains whether the function of PTBP3 in gastric carcinoma tumour formation and metastasis is related to PTBP3-regulated alternative splicing of CAV1.

In the current study, we showed that reduced PTBP3 expression inhibited the motility of MKN45 cells in vitro and suppressed tumour lymphangiogenesis and metastasis to regional lymph nodes in vivo. Finally, increased CAV1α expression with decreased PTBP3 expression was sufficient to suppress the expression and activity of steroid receptor coactivator (Src) and focal adhesion kinase (FAK) proteins following integrin engagement, suggesting that CAV1 is the downstream target of PTBP3.

## Materials and methods

### Tissue microarray and immunohistochemistry

A gastric cancer tissue microarray (HStmA180SUR07) was purchased from the Shanghai Outdo Biotech Co., Ltd. (Shanghai, China) and included 90 gastric adenocarcinoma tissues with follow-up data and 90 matched adjacent normal gastric mucosa specimens. Immunohistochemistry (IHC) was performed as previously described^18^.

### Cell culture and vector construction

Gastric cancer cells (MKN45 and SGC7901) were obtained from the Type Culture Collection of the Chinese Academy of Sciences (Shanghai, China). The cells were cultured in RPMI-1640 medium supplemented with 10% foetal bovine serum and 100 U/mL penicillin/streptomycin (Invitrogen Corporation, Carlsbad, CA, USA) in a humidified atmosphere with 5% CO_2_ at 37 °C.

A hairpin small interfering RNA (siRNA) oligonucleotide strand specific for PTBP3 siRNA was designed according to the Silencer™ neo Kit Protocol (Invitrogen, Carlsbad, CA, USA) and synthesised by Shanghai Jierui Biotech Co., Ltd. The oligonucleotide strands were diluted and annealed into double strands before insertion into the *pSilencer* 2.1-U6 vector (Invitrogen, Carlsbad, CA, USA), forming the PTBP3 siRNA expression plasmid.

Plasmids expressing PTBP3 siRNA (MKN45-KD) and *pSilencer* 2.1-U6 neo negative control plasmids (MKN45-NC) were independently transfected into MKN45 cells using Lipofectamine™ 2000 (Invitrogen, Carlsbad, CA, USA) according to the manufacturer’s instructions. After 2 days, 600 μg/mL of G418 (Invitrogen, Carlsbad, CA, USA) was used to screen cells stably expressing PTBP3 siRNA. The sequence of the PTBP3 siRNA was 5′-GAGUGAAGAUUAUGUUUAATT-3′.

The coding region of complementary DNA (cDNA) was cloned into the pcDNA 3.1(+) vector, and the constructs were verified by DNA sequencing. The following sequences were used: pcDNA (PTBP3) sense: 5′-CAAGCTTGACACCAGGGGTCTGGACTTA-3′, anti-sense: 5′-CGGGATCCTATGGTCCAGTTTTAGGAGA-3′; pcDNA (CAV1α) sense: 5′-CCGAGCGCGGCCGCCATGTCTGGGGGCAAATAC-3′, anti-sense: 5′-TATCTGGGGCCGCTTATGTTTCTTTCTGCATGTTG-3′.

### Growth curves and the colony formation assay

Growth curves and the colony formation assay were measured as previously described^24^.

### Wound-healing assay

The wound-healing assay was performed as previously described^25^.

### Invasion and migration assays

Invasion and migration assays were performed as previously described^26^.

### SDS-PAGE and western blot analysis

The following antibodies were used: CAV1α (Abcam, USA); CAV1, Src, p-Src, FAK, and p-FAK (Signalway Antibody, USA); β-actin (Protein Tech Group, China). The proteins were separated on 12% gels for CAV1α, CAV1, β-actin, Src, and p-Src and on 8% gels for FAK, p-FAK, and Integrinβ3. β-Actin was used as the internal standard.

### Real-time reverse transcription-PCR

CAV1α, CAV1β, and PTBP3 mRNA was normalised to GAPDH mRNA. The primer sequences were as follows: CAV1α, forward: 5′-CAGAACAAACCTTTGGCGGG-3′ and reverse: 5′-GGGCTTGTAGATGTTGCCCT-3′; CAV1β, forward: 5′-AGTGTCCGCTTCTGCTATCT-3′ and reverse: 5′-AAGGGAGCATCCTAGACCCA-3′; PTBP3, forward: 5′-TCTCCGTGCCTTCAGTCAGC-3′ and reverse: 5′-GCTTTACGCATCGTCACGC-3′; GAPDH, forward: 5′-CAGGGCTGCTTTTAACTC-3′ and reverse: 5′-GGAAGATGGTGATGGGAT-3′. The results were calculated using the 2^-△△CT^ method, and the results are expressed as the mean ± SD^27^.

### RNA interference

Short interfering RNAs specifically targeting PTBP3, CAV1, or CAV1α were transfected into the indicated cells at a final concentration of 100 nM using a transfection reagent (Biotool) according to the manufacturer’s instructions.

### RNA immunoprecipitation (RIP) assay

RIP experiments were performed using the Magna RIP RNA-Binding protein immunoprecipitation kit (Millipore, Bedford, MA, USA) according to the manufacturer’s instructions. The collected RNAs were subjected to reverse transcriptase (RT)-quantitative PCR (qPCR) analysis to quantify the enrichment of PTBP3. Anti-rat IgG was used as controls. The primer sequences were as follows: CAV1 sense: 5′-GGGAGAAACGTTCTCACTCG-3′, anti-sense: 5′-GGAAGTGATGGTGGCAGTTT-3′.

### Dual-luciferase reporter gene assay

Dual-luciferase reporter gene assays were performed using the Dual-Luciferase Reporter Assay System (Promega, Madison, WI, USA). Cell lysates were assayed for luciferase activity following the manufacturer’s protocol (Promega) using a Centro XS3 LB960 microplate luminometer (Berthold Technologies, Bad Wildbad, Germany). Renilla luciferase activity was normalised to firefly luciferase activity.

### Xenotransplantation and analysis of tumours

All experiments performed on animals were performed in accordance with institutional guidelines. In addition, we followed the ARRIVE guidelines^28^. Approximately, 1.0 × 10^7^ MKN45-NC and MKN45-KD cells in 100 μL of serum-free medium were implanted into the subcutaneous tissue of the right abdominal wall of female severe combined immunodeficient mice (7–8 weeks old, one tumour per mouse, *n* = 6 mice per group). The tumours were measured with callipers, and the tumour volumes were calculated as follows: volume = length × width^2^ × 0.52. The mice were sacrificed after 6 weeks, and the tumours and axillary lymph nodes were collected. The length and width of the lymph nodes were collected as volume = (π/6) × (length × width)2/3^29^. Each tumour sample was snap-frozen in liquid nitrogen or fixed immediately in 4% paraformaldehyde overnight at 4 °C and then processed for further histologic analysis^5, 30^.

### Histology

Paraffin sections (6 μm) of tumours were immunostained with antibodies against lymphatic vessel endothelial hyaluronan receptor 1 (LYVE-1, Protein Tech Group, China). Sections of the axillary lymph nodes were stained with haematoxylin and eosin.

### Gene set enrichment analysis (GSEA)

The data used for GSEA were accessible from The Cancer Genome Atlas (TCGA, https://tcga-data.nci.nih.gov/docs/publications/tcga/), Gene Expression Omnibus (GEO, http://www.ncbi.nlm.nih.gov/gds/), and were analysed using the software GSEA v2.2.2 (http://www.broadinstitute.org/gsea). The high and low groups of clinical gastric cancer specimens were separated according to the median PTBP3 expression level. Statistical significance (false discovery rate) was set at 0.25.

### Statistical analyses

Statistical analysis was performed using SPSS 18.0 software. All experiments were performed at least three times, and the results are presented as the mean ± standard deviation. Student’s *t*-test (two-tailed) was used to analyse the significance of individual groups. Survival curves were plotted using the Kaplan–Meier method and compared using the log-rank test. Correlations were determined by Pearson’s correlation. We defined a high expression level as above the median and low expression as below the median. *P*-values < 0.05 were considered significant.

## Results

### PTBP3 is upregulated in gastric cancer tissues, and high PTBP3 expression correlates with a high risk of lymph node metastasis

**Fig. 1:**
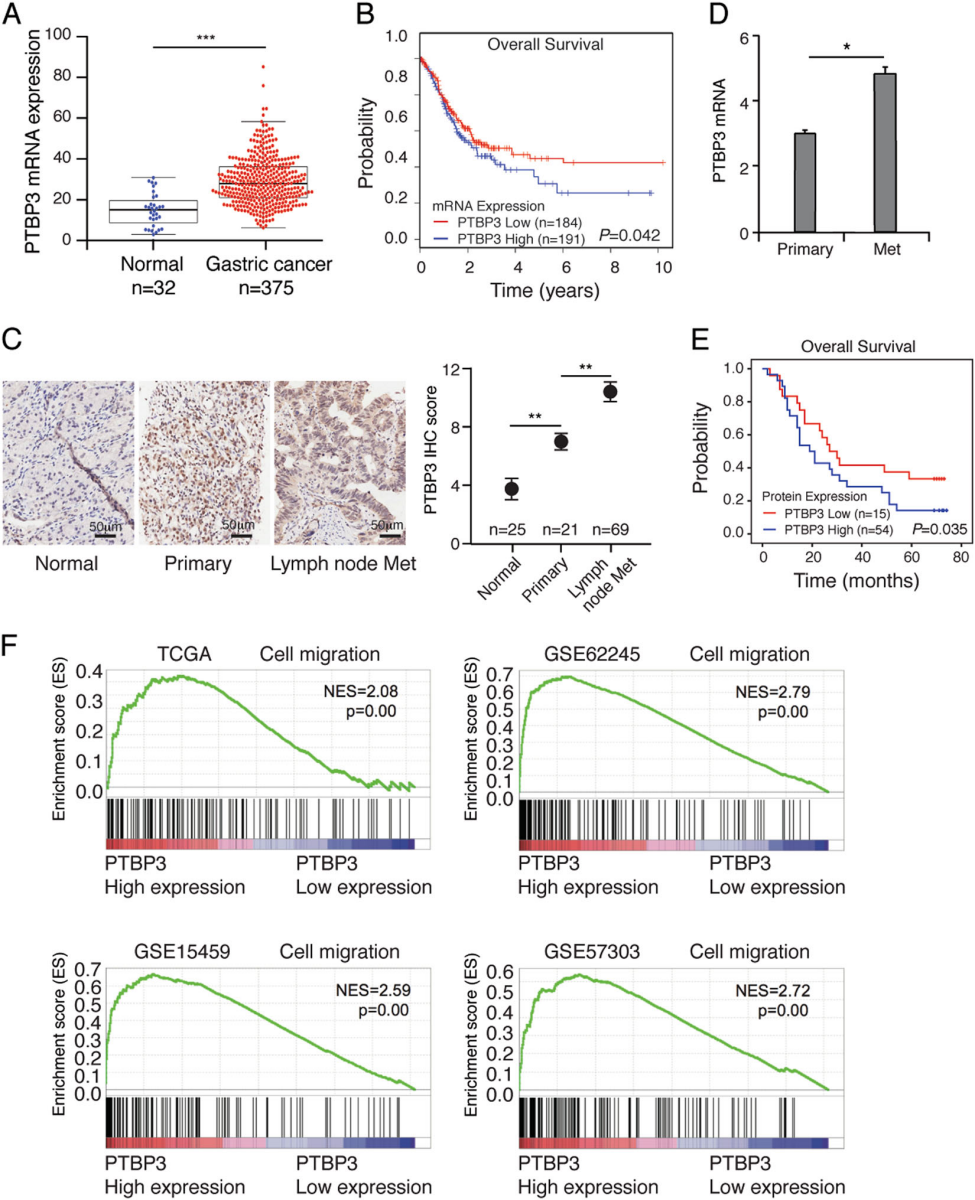
PTBP3 is upregulated in gastric cancer tissues and correlates with poor survival. **a** Dot plots represent PTBP3 expression levels in gastric cancer tissues (*n* = 375) and normal gastric tissues (*n* = 32) according to the data from the TCGA database (***P < 0.001). **b** Kaplan–Meier analysis of overall survival in patients with variable PTBP3 expression according to the data from the TCGA database (*P* = 0.042). **c** Representative images and quantitative IHC analysis of PTBP3 expression in normal gastric mucosa tissue, primary gastric cancer tissue and lymph node metastasis of gastric cancer tissue according to the clinical specimens (**P < 0.01). Scale bar, 50 μm. **d** PTBP3 mRNA levels (log2 intensity) in primary gastric cancer tissue and lymph node metastasis of gastric cancer tissue (*P < 0.05). **e** Kaplan–Meier analysis of overall survival in lymph node metastasis of gastric cancer patients with variable PTBP3 expression (*P* = 0.035). **f** High expression of PTBP3 correlated with the cell migration signalling pathway, as revealed using the TCGA, GSE62245, GSE15459, and GSE57303 datasets. NES normalised enrichment score. Each bar represents the mean ± SD of three independent experiments

To investigate the role of PTBP3 in the development of gastric cancer, the gene expression data of 375 gastric cancers were downloaded from the Cancer Genome Atlas (TCGA) database. PTBP3 expression was first analysed in 375 gastric cancer tissues and 32 normal gastric tissues, and we found that PTBP3 was significantly upregulated in gastric cancer tissues (Fig. 1a). Kaplan–Meier survival curve analysis showed that the overall survival of the patients with low levels of PTBP3 expression was obviously longer than that of patients with high levels of PTBP3 expression (Fig. 1b). These results were consistent with our previous study^31^. Our previous study also found that PTBP3 expression is associated with lymph node metastasis. To confirm these results, IHC was performed to assess PTBP3 expression in 90 gastric cancer specimens. IHC analysis and real-time PCR data showed that PTBP3 was upregulated in the tissues of patients with lymph node metastasis (Figs. 1c, d). Kaplan–Meier survival analysis showed that lymph node metastasis in gastric cancer patients with high PTBP3 expression correlated with a worse outcome than lymph node metastasis in those with low PTBP3 expression (*P* = 0.035, Fig. 1e). Moreover, GSEA comparing gastric cancer tumours with high expression and those with low expression of PTBP3 using TCGA and three independent GEO datasets showed a strong correlation between PTBP3 expression and the cell migration signalling pathway (Fig. 1f). Taken together, these findings suggest that PTBP3 is upregulated in the gastric cancer tissues of patients with lymph node metastasis, and patients with high PTBP3 expression showed a high risk of lymph node metastasis.

### Overexpression of PTBP3 promotes gastric cancer migration and invasion but has no effect on proliferation in vitro

**Fig. 2:**
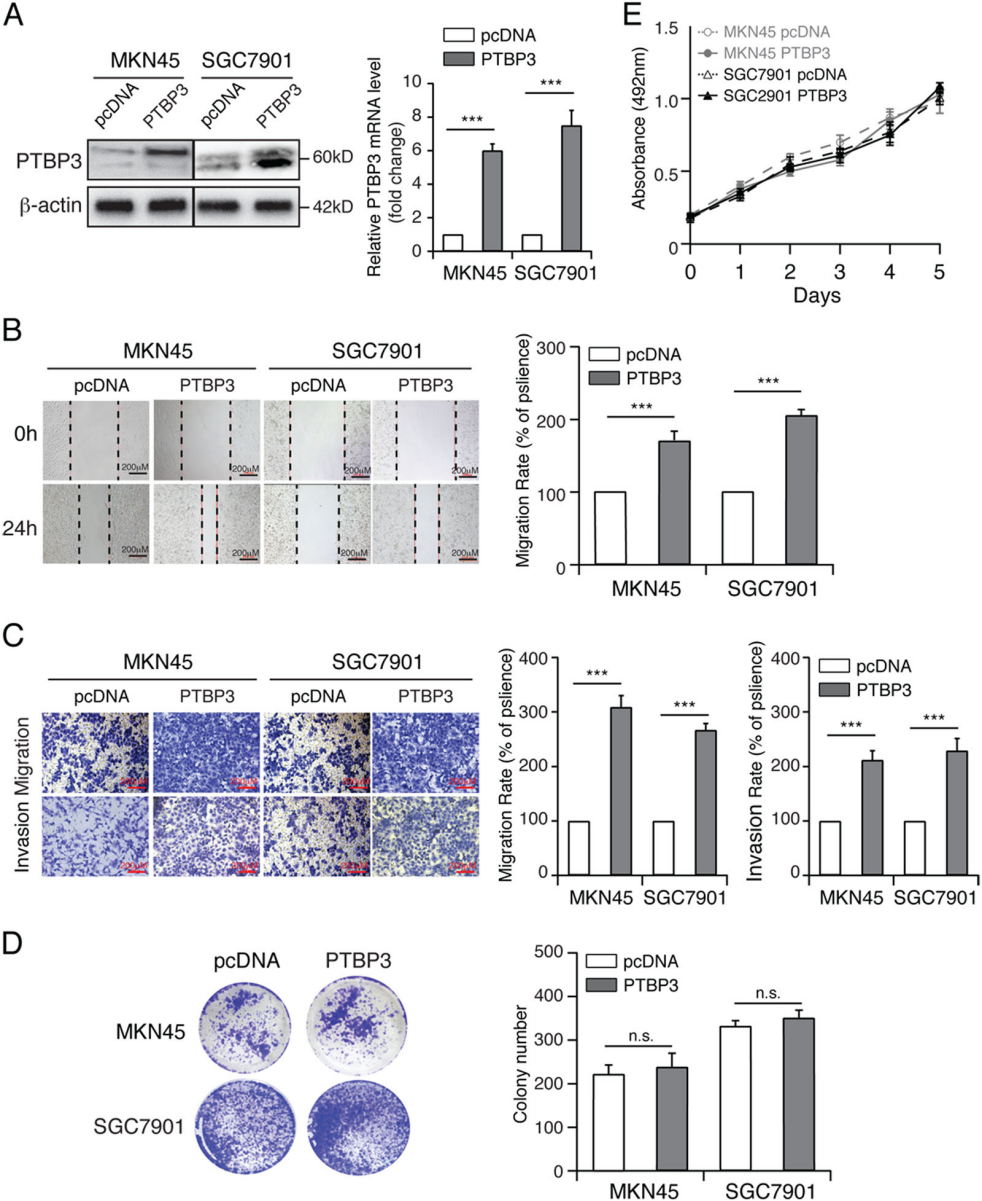
PTBP3 promotes the migration and invasion of gastric cancer cells. **a** Western blot and real-time PCR analyses of PTBP3 expression in the indicated cells with PTBP3 overexpression (***P < 0.001). **b** Wound-healing assay showing the migration ability of MKN45 and SGC7901 with PTBP3 overexpression (***P < 0.001). Scale bar, 200 μm. **c** Transwell assay showing the migration and invasion ability of the indicated cells with PTBP3 overexpression (***P < 0.001). Scale bar, 200 μm. Colony formation assay (**d**) and growth curve analysis (**e**) showing that PTBP3 overexpression had no effect on the proliferation rates of MKN45 and SGC7901. Each bar represents the mean ± SD of three independent experiments

To determine whether PTBP3 promotes gastric cancer migration and invasion, we overexpressed PTBP3 in MKN45 and SGC7901 cells using pcDNA-PTBP3 (Fig. 2a). A wound-healing assay was performed to investigate the effect of PTBP3 on the horizontal migration of MKN45 and SGC7901 cells by observing the wounded confluent monolayers of each group of cells. The wound of PTBP3-overexpressing cells was obviously narrower than that of the control cells (Fig. 2b). Therefore, PTBP3 overexpression markedly promoted the spreading of the PTBP3-overexpressing cells along the edges of the wound compared with the behaviour observed in the control cells. Transwell assays showed that PTBP3 overexpression improved the migratory ability of MKN45 and SGC7901 cells, as indicated by the increase in migrated and invaded cells (Fig. 2c).

To further determine the role of PTBP3 in regulating proliferation, we performed colony formation assays and growth curve analysis. Interestingly, the results showed no significant difference between PTBP3-overexpressing cells and control cells (Figs. 2d, e), suggesting that PTBP3 overexpression promotes gastric cancer migration and invasion but has no effect on proliferation in vitro.

### PTBP3 knockdown inhibits gastric cancer migration and invasion but has no effect on proliferation in vitro

**Fig. 3:**
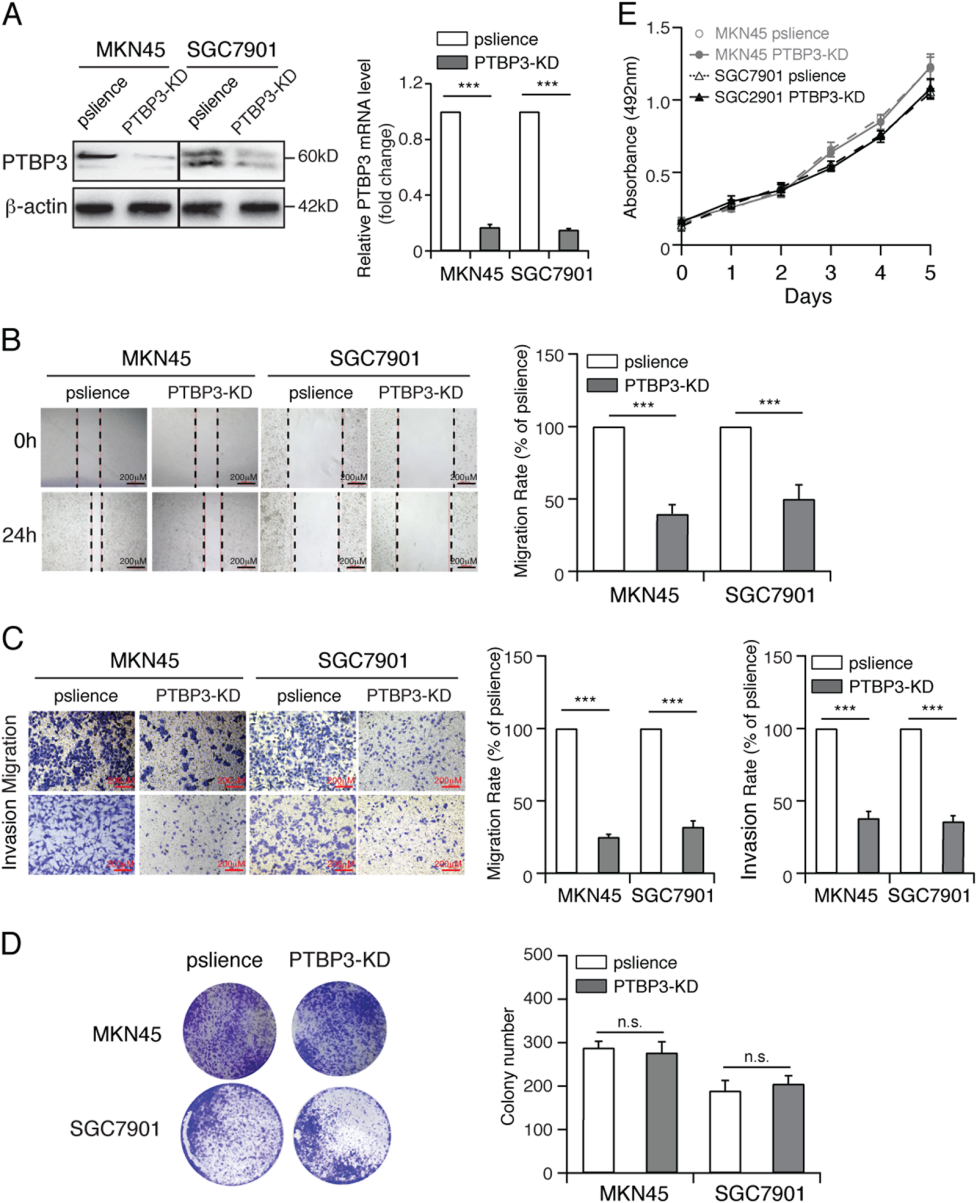
PTBP3 knockdown inhibits the migration and invasion of gastric cancer cells. **a** Western blot and real-time PCR analyses PTBP3 expression in the indicated cells with PTBP3 knockdown (***P < 0.001). **b** Wound-healing assay showing the migration ability of MKN45 and SGC7901 cells with PTBP3 knockdown (***P < 0.001). Scale bar, 200 μm. **c** Transwell assay showing the migration and invasion ability of the indicated cells with PTBP3 knockdown (***P < 0.001). Scale bar, 200 μm. Colony formation assay (**d**) and growth curve analysis (**e**) showing that PTBP3 knockdown had no effect on the proliferation rates of MKN45 and SGC7901. Each bar represents the mean ± SD of three independent experiments

To confirm the function of PTBP3 on gastric cancer migration and invasion, we silenced PTBP3 in MKN45 and SGC7901 cells using pSilencer-PTBP3 (Fig. 3a). Wound-healing and transwell assays were performed on PTBP3-knockdown MKN45 and SGC7901 cells and control cells. In the wound-healing assay, the wound of PTBP3-knockdown cells was obviously broader than that of control cells (Fig. 3b). Similarly, the transwell assay results showed that PTBP3 knockdown suppressed the migratory ability of MKN45 and SGC7901 cells, as indicated by the decrease in the migrated and invaded cells (Fig. 3c). Meanwhile, PTBP3 knockdown showed no effect on gastric cancer proliferation (Figs. 3d, e). These results confirmed the function of PTBP3 on gastric cancer migration and invasion in vitro.

### PTBP3 knockdown suppresses axillary lymph node metastasis in vivo

**Fig. 4:**
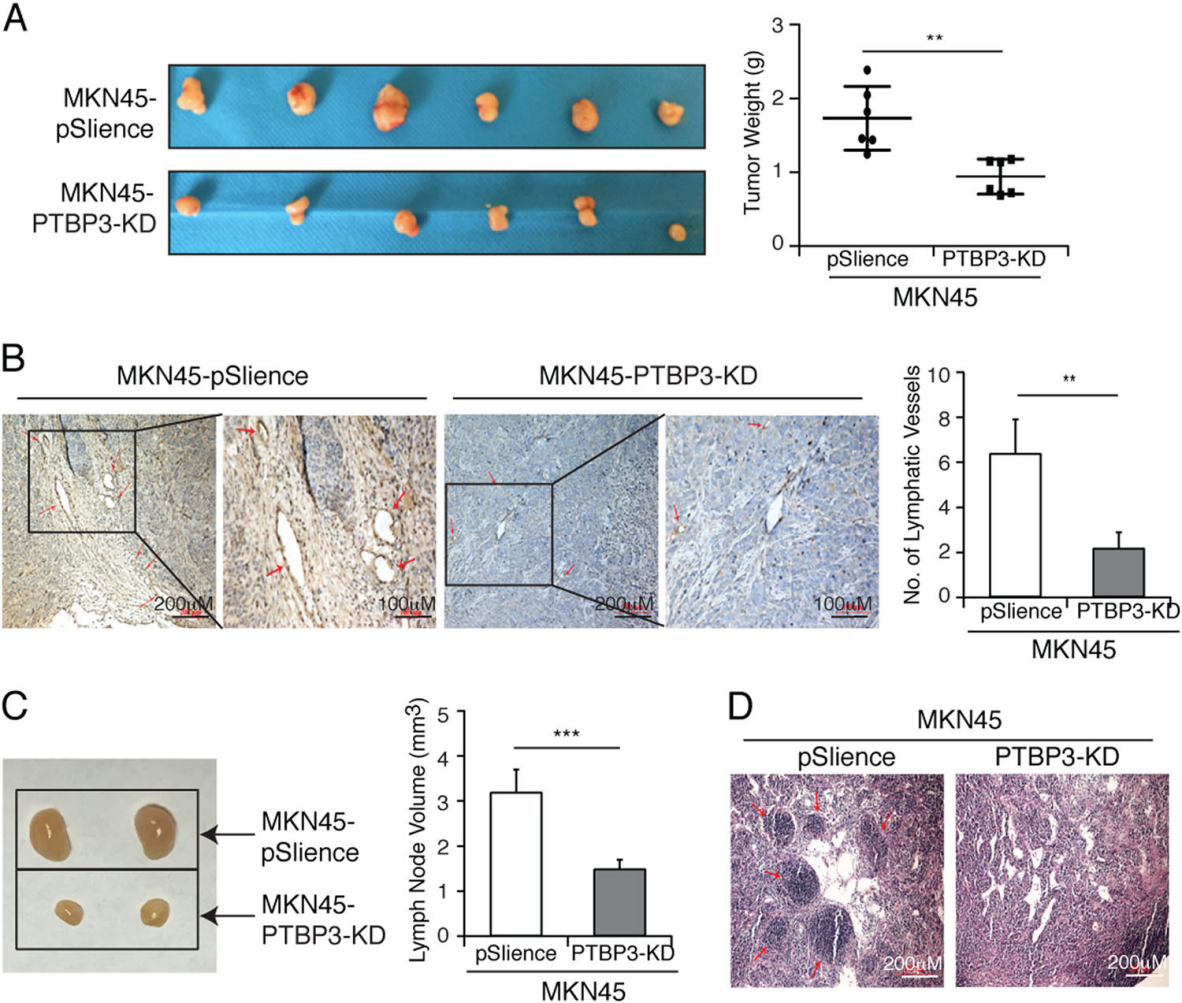
PTBP3 knockdown suppresses axillary lymph node metastasis in vivo. **a** The weight of tumours derived from the pSilence vector and PTBP3 KD-transfected MKN45 cells were calculated (**P < 0.01). **b** Immunohistochemical staining shown for LYVE-1 to identify lymphatic vessels (red arrows) at the tumour margin and LYVE-1-stained vessels in three microscopic fields of the highest vessel density were counted (**P < 0.01). **c** Typical lymph nodes in mice bearing the MKN45-PTBP KD and MKN45-pSilence tumours and lymph node volume were calculated (***P < 0.001). **d** Histologic staining of lymph node sections from mice with MKN45-PTBP KD and MKN45-pSilence tumours, respectively. Red arrows indicate the tumour cells in the lymph node. Scale bar, 200 μm

The tumourigenesis assay in female severe combined immune-deficient mice was used to confirm whether PTBP3 promoted tumour growth in vivo. As shown in Figure 4a, interestingly, the weight of tumours formed by PTBP3 knockdown was lower than that of control cell lines, suggesting that PTBP3 knockdown inhibited tumourigenesis in vivo, at least in this model. Lymphatic vessels in tumours related to lymph node metastasis were analysed by immunostaining with antibodies against the lymphatic endothelial marker LYVE-1. LYVE-1-positive vessel-like structures were found in control tumours, and hyperplastic lymphatic vessels were observed in the tumour periphery. By contrast, few LYVE-1-positive vessel-like structures were observed in PTBP3-knockdown tumours. The mean number of LYVE-1-positive vessels was determined from three microscopic fields with the highest vessel density, as shown in Figure 4b, as follows: MKN45-KD, 2.2 ± 0.75; control, 6.4 ± 1.6. Figures 4c, d show representative axillary lymph nodes found in tumour-bearing mice and typical lymph node sections stained with haematoxylin and eosin. The mean lymph node volume in the PTBP3-knockdown group was 1.45 ± 0.24 mm^3^, and that in the control group was 3.26 ± 0.45 mm^3^. One large metastatic lymph node was detected in the control group. Thus, a significant difference was found in the lymph node size between the two groups (***P < 0.001). Histologic analysis revealed that all 12 lymph nodes from the control group, but only 5 of 12 lymph nodes from the PTBP3-knockdown group, contained metastases. These results suggested that PTBP3 knockdown suppresses tumourigenesis and lymph node metastasis in vivo.

### PTBP3 binds to the CU-rich region of the CAV1 intron to downregulate CAV1α expression

To assess how PTBP3 expression contributes to the invasion and metastasis of gastric cancer, we next aimed to investigate the regulated genes of PTBP3 as an RNA-binding protein. Of these genes, CAV1 has a migration-regulating activity^21, 23^. Based on our observations on the metastatic function of PTBP3 in this study, intertwining with the importance of CAV1 in tumour metastasis, we questioned whether the metastasis function of PTBP3 in gastric cancer was attributed to its suppressive effect on CAV1α expression by alternative splicing.

**Fig. 5:**
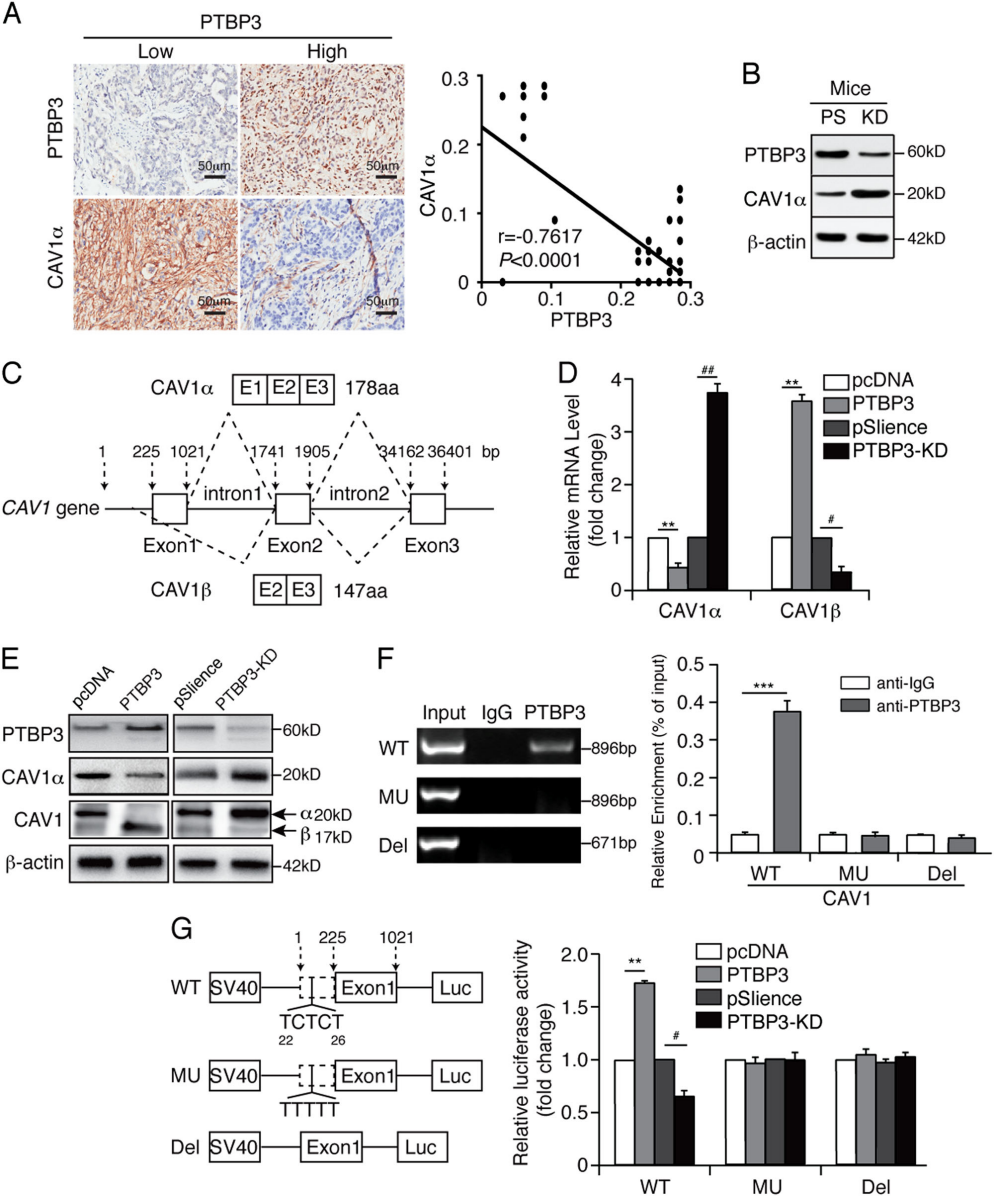
PTBP3 modulates CAV1 alternative splicing via binding to its intronic region. **a** Co-expression correlation analysis of two pairs of proteins in a clinical cohort of gastric cancer tissue samples (*n* = 69) by IHC. Images show individual protein staining in gastric cancer tissues from lymph node metastatic patients. Scale bars, 50 μm. Average cell-based staining intensity counts for each individual protein expressed in tumour cells were analysed using inForm software to assess co-expression correlations. **b** Western blot analysis of PTBP3 and CAV1α expressed in mouse tumours from PTBP3 knockdown and pSilence vector-transfected mice. **c** Schematic diagram of CAV1 alternative splicing. Real-time PCR (**d**) and western blot (**e**) analysis of the expression of PTBP3, CAV1α, and CAV1β in MKN45 cells with PTBP3 overexpression or knockdown (**P < 0.01 compared with pcDNA, ^##^*P* < 0.01 and ^#^*P* < 0.05 compared with pSilence). **f** RIP experiments were performed using the PTBP3 antibody to immunoprecipitate in total cell extracts of MKN45-WT, MKN45-MU, MKN45-Del cells, and relative enrichment was determined by reverse transcription-PCR (left panel) and quantitative real-time PCR (right panel) (***P < 0.001). **g** CAV1 WT, MU, or Del intron was inserted downstream of the SV40 promoter, and reporter activity was analysed in MKN45 cells with PTBP3 overexpression or knockdown (**P < 0.01 compared with pcDNA, ^#^*P* < 0.05 compared with pSilence). Each bar represents the mean ± SD of three independent experiments

To test our hypothesis, we analysed the expression of PTBP3 and CAV1α in gastric cancer tissues from lymph node metastatic patients. The results showed a negative correlation between the levels of PTBP3 and CAV1α protein expression (Fig. 5a). In addition, tumours from PTBP3-knockdown mice exhibited higher CAV1α expression than those from control mice (Fig. 5b). We used real-time PCR and western blotting to determine the effect of PTBP3 on the mRNA and protein levels of CAV1α and CAV1β. After transfection with pcDNA-PTBP3 or pSilence-PTBP3, CAV1α and CAV1β expression was measured in MKN45 cells. Our results showed that PTBP3 overexpression downregulated CAV1α expression but upregulated CAV1β expression and vice versa (Figs. 5c–e). RIP assays indicated that PTBP3 could combine with wild-type CAV1 mRNA (Fig. 5f), suggesting that PTBP3 downregulated CAV1α expression and upregulated CAV1β expression through alternative splicing. A fragment with a CU mutation in the intron of *CAV1* (MU), a deletion mutation in the intron of *CAV1* (Del), and wild-type *CAV1* gene (WT) were inserted into the pGL3-promoter with an SV40 promoter, which was then transfected into MKN45 cells with PTBP3 overexpression or silencing. Luciferase activity in MKN45 cells overexpressing PTBP3 was higher than that in MKN45 cells after transfection with plasmids containing the WT fragment. MKN45 cells transfected with plasmids containing the MU or Del fragment had luciferase activities that remained unchanged regardless of the PTBP3 expression level. This finding suggests that PTBP3 can interact with the WT *CAV1* gene but not with the MU *CAV1* gene (Fig. 5g) and that PTBP3 can bind to the CU-rich region to downregulate CAV1α and upregulate CAV1β expression. Our data suggest that PTBP3 downregulates CAV1α expression by binding to the CU-rich region of CAV1 intron.

### PTBP3 knockdown inhibits the migration and invasion of gastric cancer via the upregulation of CAV1α and integrin/Src/FAK pathway

**Fig. 6:**
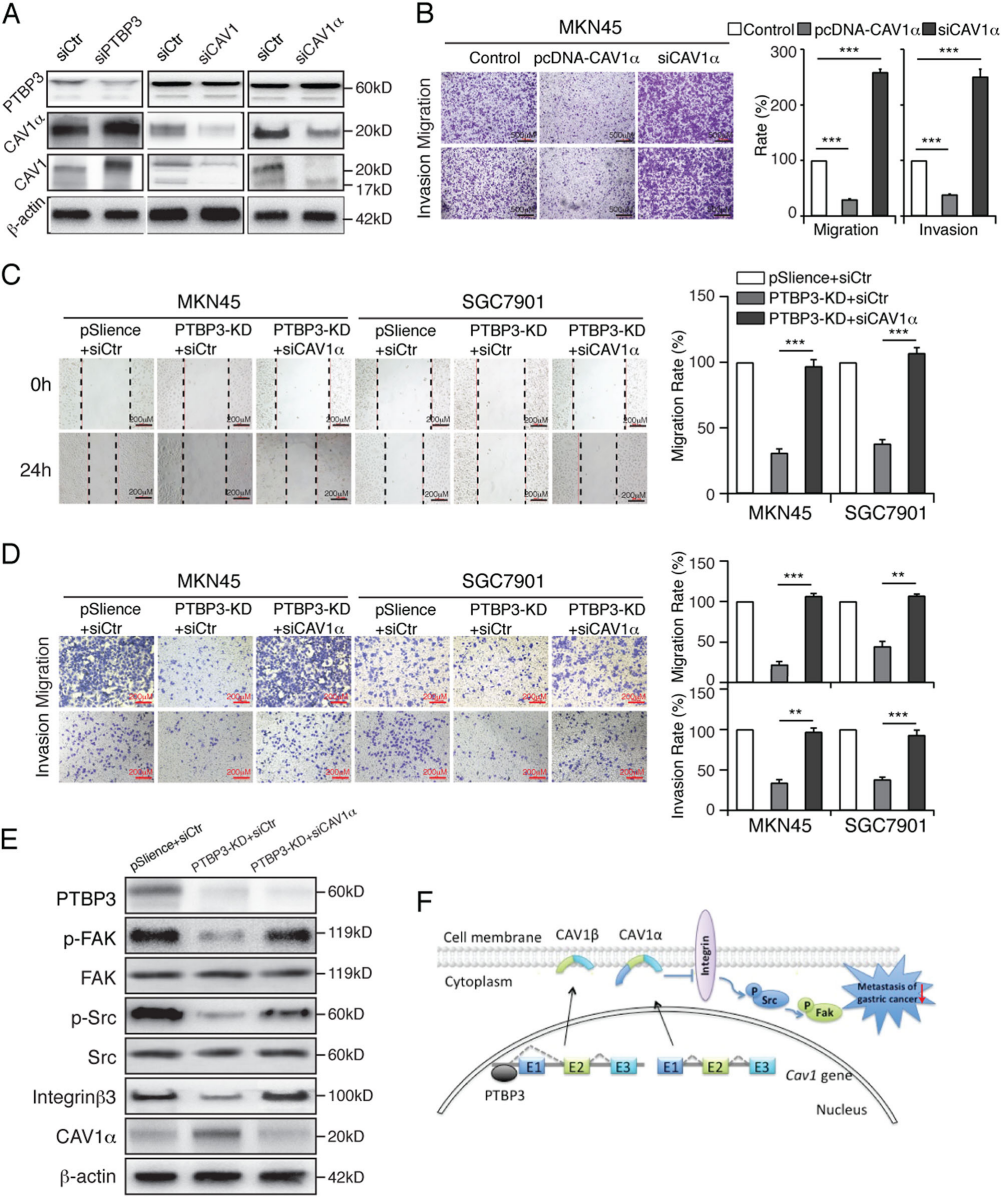
PTBP3 knockdown inhibits the migration and invasion of gastric cancer by the upregulation of CAV1α and followed the integrin/Src/FAK pathway. **a** Western blot analysis of PTBP3, CAV1, and CAV1α expressed in MKN45 cells with PTBP3 siRNA, CAV1 siRNA or CAV1α siRNA treatment. **b** Inhibition/overexpression of CAV1 affected the cell migratory ability in MKN45 cells (***P < 0.001). Scale bar, 500 μm. **c** Wound-healing assay showing the migration ability of the indicated cells. MKN45 and SGC7901 cells with PTBP3 knockdown were exposed to CAV1α-siRNA (***P < 0.001). Scale bar, 200 μm. **d** Transwell assay showing the migration and invasion ability of the indicated cells. MKN45 and SGC7901 cells with PTBP3 knockdown were exposed to CAV1α-siRNA (**P < 0.01, ***P < 0.001). Scale bar, 200 μm. **e** Western blot analysis with antibodies directed against CAV1α, FAK, p-FAK(Y397), Src, p-Src(Y418), and integrinβ3 was performed. β-Actin was used as a loading control. PTBP3 knockdown induced the reduction of integrinβ3, p-Src, and p-FAK and was abrogated by the knockdown of CAV1α in MKN45-KD cells. Each bar represents the mean ± SD of three independent experiments. **f** Schematic diagram of PTBP3 knockdown inhibiting the migration and invasion of gastric cancer by the upregulation of CAV1α through the integrin/Src/FAK pathway

To further verify the function of CAV1α in gastric cancer cell migration, we transfected with the CAV1α siRNA/overexpression plasmid into MKN45 cells (Fig. 6a). The transwell results indicated that CAV1α overexpression inhibited cell metastasis and invasion and vice versa (Fig. 6b). We further demonstrated whether PTBP3 could regulate invasion and migration by regulating CAV1α expression. The cell wound-healing assay showed the invasive ability of gastric cancer cells with PTBP3 knockdown and CAV1α inhibition was promoted (Fig. 6c). The results from the transwell assay showed the same trend (Fig. 6d).

To further elucidate the underlying mechanisms, we used western blotting to analyse the protein level of integrin/Src/FAK pathway proteins in different groups, including PTBP3-knockdown MKN45 and SGC7901 cells, PTBP3-knockdown and siCAV1α-transfected cells and control cells. We found that phosphorylated FAK(Y397) and Src(Y418) activation were increased in PTBP3-knockdown and siCAV1α-transfected MKN45 and SGC7901 cells compared with the levels in control cells (Fig. 6e). These results, however, were not associated with reduced expression of FAK/Src proteins. Overall, PTBP3 mediates CAV1 signalling through alternative splicing, and CAV1α has a negative effect on the metastasis potential of gastric cancer cells following integrin/Src/FAK pathway activation (Fig. 6f).

## Discussion

Hou et al. found that PTBP3 promotes the epithelial–mesenchymal transition in breast cancer by regulating ZEB1 mRNA stability^32^. Moreover, Chen et al. have previously reported that PTBP3 overexpression may be closely positively correlated with the progress of gastric carcinoma^18^. In the present study, we found PTBP3 to be upregulated in gastric cancer tissues of patients with lymph node metastasis. Patients with high PTBP3 expression had significantly shorter survival than those with low PTBP3 expression (Fig. 1). Overexpression/knockdown of PTBP3 expression had no effect on proliferation, whereas both affected migration and invasion in vitro (Figs. 2 and 3). In addition, consistent with our previous research^18^, when a mouse xenotransplant model of MKN45 was established, MKN45 cells with PTBP3 knockdown formed tumours that were smaller in size than their counterparts and exhibited suppressed tumour lymphangiogenesis and metastasis to regional lymph nodes (Fig. 4). Stable knockdown of PTBP3 does not affect proliferation in vitro (Figs. 2 and 3) but does inhibit tumour growth in vivo (Fig. 4). We suggest that the malignant proliferation characteristics of tumour cells in vitro were maintained because the intracellular signal transduction pathway of proliferation was generated by the compensatory mechanism after stable knockdown of PTBP3. However, in the in vivo environment, stable knockdown of PTBP3 may inhibit tumour differentiation and growth by affecting the tumour microenvironment and not only through the CAV1 pathway.

Given that our data indicate that PTBP3 behaves as a ‘metastasis-promoter gene’ in gastric cancer, we next sought to determine the possible mechanisms for this observed phenotype. Post-transcriptional mechanisms play an important role in the regulation of gene expression through a multitude of RNA-binding proteins^33, 34^. CAV1 function has been reported to be mediated by PTBP3 through alternative splicing by directly binding to CAV1 mRNA^10^; however, the detailed mechanism of how PTBP3 regulates CAV1 is unclear. Our results suggest that PTBP3 downregulates CAV1α expression by binding to the CU-rich region of the CAV1 intron, and the mRNA and protein expression levels of CAV1α are upregulated with the decrease in PTBP3. Thus, CAV1α function may be mediated, at least in part, by PTBP3 through alternative splicing (Fig. 5).

CAV1 is a 178-amino-acid protein that exists as two variants. Although both CAV1α and CAV1β are co-expressed in most human cells, only CAV1α can be phosphorylated on Tyr-14 by Src or Fyn^35^. CAV1α and CAV1β have different functions in cancer progression. Shajahan et al. reported that CAV1α promotes cell death, whereas CAV1β promotes cell survival^22^. A previous study reported that specific extracellular matrix (ECM)–integrin interactions and Tyr-14 phosphorylation are required for CAV1-enhanced melanoma cell migration, invasion, and metastasis to the lung^23^. ECM–cell interactions are important for metastatic phenotype formation. In addition, CAV1α inhibits melanoma metastasis by regulating the integrin/Src/FAK pathway^21^. However, it appears that CAV1α acts as a suppressor or enhancer of integrin/Src/FAK pathway activity depending on cell type^36, 37^.

Our results, showing a significant reduction in the activities and expression of both Src and FAK in PTBP3-KD cells with significantly increased CAV1α expression compared with control cells, are consistent with their reduced motility in vitro and their metastatic potential in vivo. Additionally, CAV1α expression in MKN45-KD resulted in a dramatic reduction in the expression of integrinβ3, a molecule implicated in regulating the motility and metastatic ability of cancer cells^38^. To determine whether CAV1α is a downstream target of PTBP3-induced MKN45 cell invasion, we knocked down CAV1α expression using siCAV1α in MKN45-KD cells. We found that the siPTBP3-induced reduction of integrinβ3, p-Src and p-FAK was abrogated by the knockdown of CAV1α (Fig. 6). These data suggest that CAV1α may be responsible for PTBP3-mediated cell invasion in MKN45 cells.

Tumour metastasis to regional lymph nodes is common in gastric cancer, and an association between lymphangiogenesis and tumour metastasis has been reported^39–41^. The spread of tumour cells to local lymph nodes is an early event in tumour metastasis^42, 43^. The results of our study show that PTBP3 expression is increased in a tumour cell line selected for lymphatic metastasis and provides direct evidence supporting the hypothesis that the inhibition of PTBP3 can block tumour lymphangiogenesis and suppress lymph node metastasis.

In summary, our study raised the possibility that the examination of PTBP3 expression by IHC could be clinically used as a tool to identify gastric cancer patients with an increased risk of tumour invasion and metastasis and provides additional evidence for PTBP3 as a metastatic gene in gastric cancer.
